# Immediate Effects of a Wearable Thermoplastic Polyurethane Arch Pad Sock on Lower-Extremity Muscle Activity in Adults with Flexible Flatfoot

**DOI:** 10.3390/s26196150

**Published:** 2026-09-28

**Authors:** Dongwoo Ha, Chang-Yoon Baek, Hyeong-Dong Kim

**Affiliations:** 1 Department of Rehabilitation Science, Graduate School of Health Sciences, Korea University (Anam), Seoul 02841, Republic of Korea; 2026021027@korea.ac.kr; 2 Department of Rehabilitation Center, National Health Insurance Ilsan Hospital, Ilsan 100, Republic of Korea; bcy77@nhimc.or.kr; 3 Graduate School of Sports Medicine, CHA University, Pocheon-si 11160, Republic of Korea; 4 Department of Health and Environmental Sciences, Korea University (Anam), Seoul 02841, Republic of Korea

**Keywords:** flatfoot, pes planus, surface electromyography (sEMG), thermoplastic polyurethane (TPU), wearable arch pad sock, immediate effect, gait

## Abstract

Flatfoot (pes planus) is a common foot deformity associated with altered lower-extremity muscle activity during gait. Conventional foot orthoses and insoles can provide arch support but have limitations related to comfort, adherence, and everyday usability. This study evaluated the immediate effects of a thermoplastic polyurethane (TPU) arch pad sock on lower-extremity muscle activity in adults with flexible flatfoot. Twenty adults with flexible flatfoot participated. Surface electromyography signals were recorded bilaterally from the tibialis anterior (TA), peroneus longus (PL), medial gastrocnemius (MG), and lateral gastrocnemius (LG), which yielded eight recording channels. Muscle activity was measured before and immediately after the TPU arch pad sock application and normalized to percentage of maximum voluntary isometric contraction. Muscle activity was significantly reduced in five of the eight channels following the TPU arch pad sock application (all *p* < 0.05), including the right TA, right PL, right MG, left TA, and left PL. The largest effect was observed in the right MG (Cohen’s d = 1.157). Although these reductions were statistically significant, all five channels remained below the minimal detectable change (MDC95), indicating that the immediate effects did not reach the threshold for clinically meaningful change; longitudinal studies evaluating cumulative use are needed to establish clinical significance. Wearing the TPU arch pad sock was immediately associated with altered lower-extremity muscle activity in the subjects, although the single-arm design precludes establishing a definitive causal attribution. These findings suggest that the device may affect neuromuscular responses during walking by providing medial longitudinal arch support.

## 1. Introduction

Flatfoot (pes planus) is characterized by partial or complete collapse of the medial longitudinal arch under weight-bearing conditions [1]. These conditions may be caused by congenital or acquired factors and are broadly classified into flexible and rigid, in terms of nature.

The global prevalence of flatfoot is approximately 15.6%, with male and Asian individuals having higher rates than female and White individuals, respectively [2].

In Korea, a nationwide study of 560,141 male conscripts conducted between 2018 and 2020 reported a flatfoot prevalence of approximately 2.9% [3]. This discrepancy between global and Korean prevalence estimates likely reflects differences in the age range and composition of the source populations—the global estimate by Salinas-Torres et al. [2] pooled data from children (including age groups 3–17 years) and adults across 12 studies, whereas the Korean estimate by Ryu et al. [3] was derived exclusively from young adult male conscripts assessed via weight-bearing radiographs—in addition to broader methodological heterogeneity across the pooled studies in [2].

Flatfoot is associated with adverse clinical outcomes in multiple lower-extremity and spinal regions. In individuals with severe flatfoot, the incidence of anterior knee pain and intermittent low back pain increases approximately two-fold [4]. In a cohort of 1930 adults, flatfoot was also identified as an independent risk factor for low back pain [5].

Compared with normal-arched gait, flatfoot is associated with characteristic kinematic deviations, including increased rearfoot eversion and forefoot abduction [6]. These deviations are secondary to excessive pronation caused by collapse of the medial longitudinal arch.

These altered mechanics produce compensatory changes in lower-extremity neuromuscular activity.

Using surface electromyography (sEMG), Murley et al. [7] identified characteristic alterations in lower-extremity muscle recruitment during flatfoot gait. They found an approximately 19% increase in tibialis anterior (TA) activity and an approximately 13% decrease in peroneus longus (PL) activity during the contact phase [7].

Accordingly, in this study, the following four lower-extremity muscles reported to exhibit activity abnormalities during flatfoot gait were selected as the primary measurement targets: TA, PL, medial gastrocnemius (MG), and lateral gastrocnemius (LG).

Previous studies have shown that prefabricated insole use can partially normalize aberrant muscle activity patterns during flatfoot gait [8]. This finding suggests that appropriate assistive device intervention represents a viable strategy for neuromuscular management in this population. However, a systematic review found insufficient evidence to support the efficacy of conventional insoles in reducing pain and fatigue in adults with flatfoot [9].

Moreover, adherence to prescribed orthotic devices remains poor, with 6–80% patients reporting noncompliance due to pain, discomfort, and aesthetic concerns [10]. These shortcomings underscore the need for novel assistive device designs to better accommodate the demands of daily life.

Sock-type assistive devices have emerged as promising alternatives owing to their high daily-life applicability. Specifically, they require neither prescription nor custom fabrication and can be worn indoors independently of footwear.

However, the arch support structures of most commercially available flatfoot socks are produced from the same fabric as the sock body, or from silicone. These materials cannot provide sufficient resistance against medial longitudinal arch collapse under weight-bearing conditions.

Therefore, a sock-type device incorporating a rigid arch support structure capable of sustaining adequate corrective force under weight-bearing conditions is needed.

Among rigid materials, thermoplastic polyurethane (TPU) exhibits excellent elasticity and durability and is widely adopted in foot orthotics and rehabilitation applications [11]. These properties make TPU a sustainable candidate for this application. To our knowledge, effects of a wearable assistive device integrating a TPU arch pad within a sock have not been previously studied. Therefore, this study was conducted as an initial exploratory investigation to address this gap.

This study aimed to evaluate the immediate effects of the developed TPU arch pad sock on lower-extremity muscle activity during gait in adults with flexible flatfoot. This sock combines the daily-life applicability of a sock-type device with the structural arch support function of a TPU pad. We hypothesized that application of the TPU arch pad sock significantly alters lower-extremity muscle activity, which reflects enhanced biomechanical support of the medial longitudinal arch.

## 2. Methods

This study employed a single-group pre–post experimental design to evaluate the immediate effects of the developed wearable TPU arch pad sock on lower-extremity muscle activity in adults with flexible flatfoot.

### 2.1. Participants

Twenty adults with flexible flatfoot (ten males and ten females) participated in this study.

The participant demographic information was collected using a self-reported questionnaire before testing. The participants had a mean age of 25.4 ± 2.4 years, height of 167.6 ± 6.7 cm, body mass of 66.6 ± 12.9 kg, body mass index (BMI) of 23.60 ± 3.60 kg/m^2^, and foot length of 251.5 ± 14.9 mm.

The participants were recruited using social media advertisements following institutional review board approval. Flexible flatfoot was assessed using the navicular drop test [12]. The participants were classified as having flexible flatfoot when the difference between the navicular heights in non-weight- and weight-bearing positions was ≥10 mm.

The eligible participants were healthy adults aged 20–29 years with clinically confirmed flexible flatfoot who were able to perform independent daily activities and normal gait. Additional eligibility criteria included a foot size between 230 and 270 mm and no regular use of corrective insoles, orthoses, or functional socks. The participants were excluded if they had the following:

- musculoskeletal disorders other than flexible flatfoot,
- neurological disorders,
- vestibular dysfunction,
- balance impairments affecting gait,
- a history of musculoskeletal surgery within the previous six months,
- skin disorders of the foot or lower extremities,
- allergy to compression garments,
- difficulty wearing the study sock,
- foot size outside the specified range, or
- any condition deemed unsuitable by the principal investigator for safety reasons.

This study was approved by the Korea University Institutional Review Board (KUIRB-2026-0261-01). Written informed consents were obtained from all the participants prior to participation. The participants received KRW 20,000 as compensation for their participation.

The sample size (*n* = 20) was determined considering the exploratory design of this study. In exploratory studies, the sample size is determined by the feasibility of the research objectives and practical considerations rather than statistical power [13]. In this study, the sample size was set with reference to a previous EMG study on flatfoot gait ([7]: *n* = 30). The absence of a priori power analysis is a limitation of this study and further discussed in Section 4.5.

The exploratory design and sample size of this study were intended to establish feasibility and generate preliminary effect estimates to guide the design and sample size calculation of future confirmatory studies [13], rather than to test a fully powered confirmatory hypothesis. Feasibility in this study was defined as successful bilateral EMG data collection and detection of a consistent within-session directional change, both of which were achieved. It should also be noted that unilateral flatfoot confirmation may affect the interpretation of the left-side EMG findings, as the non-dominant foot’s flatfoot status was based on self-report rather than quantitative assessment (see Section 4.5).

### 2.2. TPU Sock Prototype

The TPU arch pad sock was developed for improving flatfoot gait and supporting the medial longitudinal arch under weight-bearing conditions in adults with flexible flatfoot (Figure 1).

The sock body was fabricated in an ankle sock form, using a polyester–spandex blended material, in a single size accommodating a foot length range of 230–270 mm. The arch pad was fabricated from a commercially available TPU sheet (thickness: 1 mm, color: yellow transparent, general industrial grade). TPU, which possesses a block copolymer structure of alternating hard and soft segments, exhibits excellent elasticity and durability. Owing to these properties, it is used in foot orthotics and rehabilitation applications [11].

Specifically, one flat sheet was placed as the base and another sheet was bent into a dome shape and supported on both sides by 1 mm sheet pieces, thereby resulting in a dome-shaped pad measuring 7 cm × 2.5 cm. The pad dimensions were designed based on the standard anatomical location and range of the navicular tuberosity reported in previous studies [12,13,14,15]. This prototype design range was selected to sufficiently cover the target area while minimizing compression to the surrounding anatomical structures.

The maximum height of the pad was 4.3 mm, which corresponded to approximately 35% of the mean Navicular drop (12.35 ± 2.41 mm) of the participants in this study. This height was determined with the design intent of partially supporting the medial longitudinal arch without imposing additional mechanical burden from excessive correction under weight-bearing conditions.

Each edge was smoothed using sandpaper, and each sheet was bonded using a shoe adhesive (VT-105, V-tech, Vital Technical SDN. BHD., Rawang, Malaysia). Prior to application, the adhesive was cured for three days in a well-ventilated environment to ensure sufficient evaporation of volatile components.

The pad position corresponded to the area beneath the navicular tuberosity, which represents the apex of the medial longitudinal arch. Specifically, the pad was positioned 6 cm anterior to the heel seam of the sock and 1.5–2 cm medial to the inner seam of the sock. The pad placement was determined based on the standard anatomical location of the navicular tuberosity reported in previous studies [12,13,14,15], and the pad was applied identically to all the participants. The pad was applied to both left and right socks to support the bilateral medial longitudinal arches.

In flexible flatfoot, excessive collapse of the medial longitudinal arch under weight-bearing conditions increases pronation, thereby resulting in lower-extremity alignment imbalance and reduced gait efficiency [7]. Furthermore, abnormal EMG patterns in flatfoot are altered toward normal-arched patterns by foot orthosis application [8]. Therefore, supporting the plantar surface of the medial longitudinal arch with TPU is expected to reduce the arch collapse during gait and induce a pronation correction effect. Because TPU exhibits excellent elasticity and pressure distribution properties [11], it is expected to provide continuous support function against repetitive loading during gait.

A fabric layer of the same material as the sock was stitched over the TPU pad to minimize adverse skin reactions. This layer prevented direct contact between the pad and skin.

Prior to the study, one pilot participant performed gait while wearing the sock. A visual analogue scale score of zero was obtained, indicating no adverse reactions associated with short-term use.

TPU is widely used in medical devices, and medical-grade TPU has been reported to exhibit biocompatibility according to the ISO 10993 standard [16,17]. However, the TPU used in this study was general industrial grade rather than medical grade, and this limitation is discussed in Section 4.5.

### 2.3. EMG Recording

sEMG signals were recorded before and after the TPU arch pad sock application to evaluate lower-extremity muscle activity changes associated with flexible flatfoot. Recordings were obtained using a Delsys Trigno wireless sEMG system (SP-W06, Delsys Inc., Natick, MA, USA).

Eight channels of sEMG signals were simultaneously recorded at a sampling frequency of 1925.9 Hz.

The muscles measured were the bilateral TA, PL, MG, and LG, which were selected based on previous studies on abnormal muscle activity patterns during flatfoot gait [7,8].

Electrode placement was according to the Surface Electromyography for the Non-Invasive Assessment of Muscles guidelines [18]. The electrodes were placed over the muscle belly of each muscle, and skin preparation including alcohol wiping and drying was performed prior to attachment to minimize skin impedance.

Maximum voluntary isometric contraction (MVIC) was measured prior to gait measurements for signal normalization. The MVIC measurement positions for each muscle were as follows: dorsiflexion in a seated position for TA, combined plantarflexion and eversion in a seated position for PL, and plantarflexion in a standing position for MG and LG.

The MVIC was measured against a manual resistance applied by a researcher, with three repetitions of 5 s maximum contractions per muscle and 60 s of rest between trials.

The MVIC peak value of each muscle was calculated as the maximum root mean square (RMS) value within a 1 s sliding window (50 ms step). This calculation was applied to the raw EMG signals from the three trials [19].

Gait was measured on a 10 m flat surface in the laboratory. The participants walked this course at a self-selected walking speed, with a total gait measurement duration of approximately 1 min.

Self-selected walking speed was chosen to ensure ecological validity under everyday gait conditions. Given the within-subject design of this study, walking speed variation between the pre- and post-measurements within a single session was expected to be minimal.

The gait was measured for three laps under each condition before and after the TPU arch pad sock application. The first and last 5 s of each measurement were excluded from the analysis to remove the effects of acceleration and deceleration phases.

The collected raw EMG signals were postprocessed offline using Python 3 (NumPy, SciPy libraries). Signals were output according to the hardware specifications of the Delsys Trigno system, which applies hardware band-pass filtering (bandwidth 20–450 Hz).

The RMS values were calculated over the stable gait interval (excluding the first and last 5 s) using a 200 ms sliding window with a 50 ms step [20]. They were converted to %MVIC values using the calculated MVIC peak value for normalization [19].

### 2.4. Experimental Procedure

The experimental procedure consisted of the following steps in order: screening, flatfoot diagnosis, electrode placement, MVIC measurement, and pre- and post-sock gait measurements (Figure 2).

Before the experiment, all the participants were explained the purpose and procedures of this study and completed a written informed consent form. Subsequently, the participants removed their socks to exclude the effects of external factors on foot adaptation and to accurately evaluate flatfoot state.

Flatfoot presence was assessed on the dominant foot of each participant using the navicular drop test on the day of the experiment.

The vertical height of the navicular tuberosity was measured under non-weight bearing (subtalar joint in neutral seated) and weight-bearing (natural standing) conditions. The difference between the two positions was quantified using a ruler.

The participants were classified as having flexible flatfoot if the difference between the vertical heights in the two positions was 10 mm or greater [12].

The electrode placement and MVIC measurements were in accordance with the procedures described in Section 2.3. After completion of the MVIC measurements, a 1 min rest period was provided before the gait measurements to minimize the effect of muscle fatigue.

The gait was measured under two conditions using identical protocols: baseline measurement without sock application (pre-gait) and measurement following the TPU arch pad sock application (post-gait).

Under each condition, the participants walked the 10 m flat course at a self-selected walking speed for three laps, and the detailed gait measurement procedures are described in Section 2.3. Given that the focus of the study was on immediate effects, the measurement order was fixed (pre-gait → post-gait). Post-intervention gait assessment was conducted immediately following the sock application, without a designated adaptation period. The total experiment duration per participant was approximately 30–60 min.

### 2.5. Statistical Analysis

All the statistical analyses were performed using IBM SPSS Statistics (version 30.0.0.0; IBM Corp., Armonk, NY, USA), with the significance level set at α = 0.05, and all the variables were expressed using mean and standard deviation (Mean ± SD).

Descriptive statistics including mean, SD, minimum, and maximum were calculated for the %MVIC values of each channel.

For all statistical analyses, each participant’s EMG activity was calculated as the mean %MVIC across three gait trials per condition; therefore, N = 20 refers to the number of participants, not the number of individual trial observations (20 participants × 3 trials = 60 observations per condition).

The Shapiro–Wilk test was performed to assess the normality of the difference between the values (delta) of the pre- and post-gait measurements. Because the Shapiro–Wilk test is an appropriate normality test for studies with a sample size of 50 or smaller [21], appropriate statistical tests were performed according to the normality test results for each channel.

To control Type I error over the eight channels, the false discovery rate (FDR) was controlled using the Benjamini–Hochberg procedure [22].

For the channels following a normal distribution (*p* > 0.05), the paired *t*-test was conducted, and the effect size was calculated as Cohen’s d [23].

For the channels not following a normal distribution (*p* < 0.05), the Wilcoxon signed-rank test was performed, and the effect size was calculated using the formula r = |Z|/√N [24].

The effect size criteria for Cohen’s d were set as small (≥0.2), medium (≥0.5), and large (≥0.8) and for the Wilcoxon r as small (≥0.1), medium (≥0.3), and large (≥0.5) [23,24].

Within-trial reliability of EMG activity was assessed using intraclass correlation coefficient (ICC) (3,1) based on a two-way mixed model with consistency criteria [25]. Reliability was assessed using only the pre-intervention gait trial for each participant, as this trial provided a continuous recording suitable for internal segmentation. The stable gait interval (excluding the first and last 5 s) was divided into three equal time-based segments; for each segment, EMG activity was computed as the mean %MVIC using the same RMS and normalization procedures described above. These three within-trial segment values were entered as repeated measurements in the ICC(3,1) analysis. This approach was adopted as a methodological choice to obtain repeated measurements within a single gait trial, given that muscle activity was recorded only once under each condition (pre and post), analogous to the split-half approach used to estimate internal consistency when repeated administration of a measure is not feasible [26]; accordingly, the resulting ICC reflects within-trial (intra-session) consistency of the pre-intervention measurement rather than between-day test–retest reliability, and does not, by itself, establish that the observed pre-to-post changes were caused by the intervention. As a sensitivity analysis, ICC(3,1) was additionally computed using all six segment values (three from the pre-intervention trial and three from the post-intervention trial) as repeated measurements; these combined estimates, reported alongside the primary pre-only estimates in this analysis (Table 3), remained in the good-to-excellent range (0.816–0.989) across all eight channels. Because the intervention was expected to produce genuine pre-to-post change, this combined estimate is conceptually conservative and should not be interpreted as evidence of reduced measurement reliability; it is presented to demonstrate that reliability remained acceptable even under this conservative combined approach. The ICC value criteria were set as poor (<0.50), moderate (0.50–0.75), good (0.75–0.90), and excellent (>0.90) [27].

MDC95 for each channel was calculated as MDC95 = SEM × 1.96 × √2, where the standard error of measurement (SEM) was derived as SEM = SD(pre) × √(1 − ICC), using the pre-intervention standard deviation and the corresponding channel-specific ICC(3,1) value, following the approach of Weir [28].

This study was an exploratory investigation evaluating the immediate effects of the TPU arch pad sock, and a priori power analysis was not performed [13].

## 3. Results

### 3.1. Demographic Characteristics

A total of 20 adults with flexible flatfoot (ten males and ten females) participated in this study. The participant demographic characteristics are listed in Table 1.

Based on Table 1, the mean age of the participants is 25.4 ± 2.4 years, height is 167.6 ± 6.7 cm, weight is 66.6 ± 12.9 kg, BMI is 23.60 ± 3.60 kg/m^2^, and foot length is 251.5 ± 14.9 mm.

Flatfoot diagnosis (navicular drop test) is performed on the dominant foot of each participant, with a dominant foot distribution of 17 right feet (85.0%) and 3 left feet (15.0%). All the participants self-reported bilateral flatfoot presentation; however, quantitative assessment of the non-dominant foot was not performed, which is a limitation of this study and further discussed in Section 4.5.

The mean navicular tuberosity heights in the weight- and non-weight-bearing positions are 28.80 ± 5.76 mm and 41.15 ± 6.16 mm, respectively. The mean difference between the navicular tuberosity heights at the two positions (navicular drop, ND) is 12.35 ± 2.41 mm (range: 10.00–19.00 mm). All the participants met the flatfoot classification criterion of 10 mm or greater [12].

### 3.2. EMG Activity

The Shapiro–Wilk test results and pre- and post-intervention EMG activity (%MVIC) for all the eight channels are listed in Table 2.

Statistically significant reductions in muscle activity were observed in five of the eight channels following the sock application. Among the channels meeting the normality assumption, significant reductions (*p* < 0.05) were observed in the right PL (t = 4.031, *p* = 0.001, d = 0.901), right MG (t = 5.175, *p* < 0.001, d = 1.157), and left TA (t = 3.064, *p* = 0.006, d = 0.685).

Among the channels not meeting the normality assumption, significant reductions were observed in the right TA (Z = −4.130, *p* < 0.001, r = 0.924) and left PL (Z = −2.166, *p* = 0.030, r = 0.484).

No significant changes were observed in the right LG (Z = −0.384, *p* = 0.701, r = 0.086), left MG (t = 1.241, *p* = 0.230, d = 0.278), and left LG (Z = −0.055, *p* = 0.956, r = 0.012).

Overall, statistically significant reductions in EMG activity Were observed in five of the eight channels (62.5%) following the sock application. These include three of the four right-foot channels (TA, PL, and MG) and two of the four left-foot channels (TA and PL).

Following FDR correction using the Benjamini–Hochberg procedure [22], all the five channels remained statistically significant (adjusted *p* < 0.05), thereby confirming that the results are robust to multiple comparison correction.

Pre- and post-intervention mean EMG activity comparisons over the eight channels are shown in Figure 3.

### 3.3. Measurement Reliability

The ICC(3,1) values for the pre- and post-intervention EMG activities over all the eight channels are listed in Table 3. All the eight channels show ICC values of 0.95 or above (range: 0.958–0.996), thereby indicating excellent within-trial reliability according to the criteria of Koo and Li [27]. As a sensitivity analysis, ICC(3,1) values combining pre- and post-intervention segments are also presented in Table 3; all channels remained in the good-to-excellent range (0.816–0.989).

### 3.4. Minimal Detectable Change at 95% Confidence Level vs. Delta

The mean change (|Δ|) of the five statistically significant channels was compared with the MDC95 of each respective channel.

The comparison revealed that the mean change was below the MDC95 of each channel in all five channels: right TA (|Δ| = 2.54 < MDC95 = 3.26 %MVIC), right PL (|Δ| = 2.67 < 5.70 %MVIC), right MG (|Δ| = 2.08 < 2.20 %MVIC), left TA (|Δ| = 1.97 < 3.96 %MVIC), and left PL (|Δ| = 0.45 < 1.64 %MVIC). The |Δ| and MDC95 comparison results for all the five channels are listed in Table 4.

These findings reflect the inherent characteristics of immediate effect measurements and indicate that the changes observed within a single-session intervention do not reach the level of clinically meaningful change exceeding measurement error.

## 4. Discussion

### 4.1. Principal Findings

This study evaluated the immediate effects of the TPU arch pad sock application on lower-extremity muscle activity during gait in adults with flexible flatfoot. Statistically significant reductions in EMG activity were observed in five of the eight channels following the sock application, and measurement reliability was excellent over all the channels. However, these findings should be interpreted with appropriate caution given key methodological constraints of the study design: the absence of a control or sham-sock condition, the fixed pre-to-post measurement order, and the lack of quantitative monitoring of walking speed (see Section 4.5), any of which could have contributed to the observed changes independent of the TPU arch pad sock itself.

Among the five channels in which statistically significant changes were observed following sock application, large effect sizes were observed in three channels: right TA, right PL, and right MG. Medium effect sizes were showed in two channels: left TA and left PL. In terms of channel distribution, significant reductions were observed in three of the four right-foot channels and two of the four left-foot channels.

All the eight channels showed ICC values ≥ 0.95, thereby confirming excellent within-trial reliability. However, mean changes in all the five significant channels were below their respective MDC95 thresholds, which indicated that the observed effects did not attain the level required for clinically meaningful change.

In this study, clinical significance was operationally defined using MDC95, which represents the smallest change in EMG activity that exceeds measurement error at the individual level; changes below this threshold, although potentially reflecting a real biological effect, cannot be distinguished from measurement variability with 95% confidence.

It should be noted that MDC95 values were derived from test–retest reliability data collected under repeated measurement conditions; as such, they may represent a conservative benchmark when applied to single-session immediate effects, where within-session physiological variability differs from between-day variability. This suggests that the failure to exceed MDC95 in the present study may partly reflect the conservative nature of the benchmark rather than the complete absence of a meaningful immediate effect.

It should also be noted that MDC95 is conceptually distinct from the minimal clinically important difference (MCID). MDC95 is a measurement-error-based threshold indicating whether an observed change exceeds the precision of the instrument, whereas MCID reflects the smallest change that patients themselves perceive as meaningful or beneficial. The present study did not assess patient-reported or clinically anchored outcomes; therefore, MDC95 should not be interpreted as a surrogate for MCID, and no claim regarding clinical meaningfulness in the patient-centered sense is made in this study.

As this study evaluated only immediate, single-session effects, the present data cannot be used to quantitatively estimate the magnitude of change expected with cumulative or long-term sock use. Establishing dose–response relationships between duration of use and EMG activity change is an important direction for future longitudinal research.

Among the three non-significant channels (right LG, left MG, and left LG), the attenuated responses in the left MG and left LG may reflect non-dominant foot characteristics. This interpretation is supported by the fact that 85.0% of participants were right-foot dominant.

In the case of right LG, the limited immediate change may be attributable to the primary role of LG in lateral stabilization during the propulsive phase of gait. This may be less responsive to the immediate changes induced by a single-session arch support intervention. These channel-specific response differences require further investigation by gait phase-specific and subgroup analyses based on dominant foot status in future studies.

These findings suggest that the TPU arch pad sock can consistently decrease lower-extremity muscle activity in adults with flexible flatfoot. However, caution is required in interpreting the clinical significance of these results, considering these are inherent characteristics of single-session immediate effect measurement.

### 4.2. Comparison with Previous Studies

The direction of baseline EMG activity in the participants of this study was consistent with the direction of muscle activity characteristic of flatfoot gait reported by Murley et al. [7]. However, direct numerical comparison between the two studies was limited due to differences in the analysis interval. The present study used the mean %MVIC over the entire gait interval, whereas Murley et al. [7] analyzed a specific contact-phase interval. The comparison is indirectly supporting that the participants of this study comprised a sample group exhibiting muscle activity characteristics specific to flatfoot.

Previous studies evaluating the effects of arch-supportive insoles in adults with flatfoot showed directions of muscle activity change similar to those observed in this study.

Murley et al. [8] reported that prefabricated foot orthosis application in adults with flatfoot resulted in an approximately 19% reduction in tibialis posterior activity during the contact phase, whereas a customized orthosis resulted in a 12% reduction. Scott et al. [29] reported statistically significant changes in TA, PL, and MG activity in adults with flatfoot (*n* = 28) during shod walking including stability running shoes (*p* < 0.05). Furthermore, Huang et al. [30] reported changes in lower-extremity EMG parameters (median frequency) during inclined gait with arch-supportive insole use in adults with flatfoot.

The reduction in EMG activity in five of the eight channels following the sock application in this study is consistent in direction with previous studies. Those studies reported that wearable assistive devices providing arch support function alter lower-extremity muscle activity in adults with flatfoot, despite differences in device form.

The observed laterality of significant reductions—three of the four right-foot channels versus two of four left-foot channels—is more plausibly attributable to the right-dominant composition of the participant sample (85.0%). This pattern is unlikely to reflect a true difference in device efficacy between limbs. Therefore, it requires prospective verification in samples with balanced dominant foot distributions.

In summary, the changes in lower-extremity EMG activity observed following the sock application in this study are consistent in direction with previous studies. These prior studies similarly reported EMG effects of flatfoot assistive devices. This suggests that sock-type arch-supportive assistive devices may affect lower-extremity muscle activity reduction in adults with flatfoot. A summary comparison of these findings with the present study is presented in Table 5.

### 4.3. Theoretical and Clinical Implications

The EMG activity of five of the eight channels observed in this study showed a uniform decreasing pattern in the same direction. Large effect sizes were confirmed in three of these channels—right TA, right PL, and right MG—according to the Cohen criteria [23]. The uniform decreasing pattern and large effect sizes suggest that the changes observed in this study may be a systematic effect attributable to the sock application. The failure to reach MDC95 reflects the inherent characteristics of single-session intervention. The simultaneous confirmation of statistical significance and effect size may be regarded as evidence supporting the existence of a change tendency independent of the measurement error. However, this interpretation does not imply that the level of clinically meaningful change is reached, and verification through long-term cumulative effect studies is required.

Based on the a priori hypothesis, reductions in TA and PL activity with increase in MG and LG activity were expected. This hypothesis was based on the biomechanical inference that the gastrocnemius muscles may be chronically underactive in individuals with flexible flatfoot. Arch support provided by the sock was expected to facilitate normalization of gait patterns.

However, the measurement results revealed that right MG showed a statistically significant decrease with the largest effect size (d = 1.157), which is a finding in the direction opposite to the a priori hypothesis. These results suggest that MG and LG activity patterns in adults with flatfoot may not be uniform in an underactive state. Instead, these muscles may function in an overactive state as compensation for inefficient propulsion during gait. This is consistent in direction with previous studies reporting muscle activity reduction patterns following insole or orthosis application in adults with flatfoot [8].

This compensatory overactivity interpretation is consistent with the broader biomechanical understanding that flatfoot gait imposes increased eccentric and stabilizing demand on the gastrocnemius during late stance, potentially requiring elevated neuromuscular drive to control excessive pronation; a reduction in this compensatory drive following arch support application would therefore represent a favorable, rather than paradoxical, biomechanical response.

This channel-specific heterogeneity may reflect the differing biomechanical roles of the assessed muscles. The TA and PL contribute directly to medial longitudinal arch stabilization and rearfoot control, functions that are mechanically coupled to the arch-support geometry of the TPU pad; their activity may therefore respond more consistently to the intervention across limbs. In contrast, the gastrocnemius primarily drives propulsion during late stance, a function less directly modulated by arch geometry, which may explain the more heterogeneous response across the four gastrocnemius channels. The significant reduction observed specifically in the right MG, combined with the right-dominant composition of the sample (85.0%), suggests that the dominant limb, which bears greater propulsive and postural demand during single-limb support, may be more responsive to the compensatory unloading effect of arch support than the non-dominant limb. This interpretation remains hypothesis-generating and requires verification through stratified analyses by dominant-foot status in future studies.

Therefore, the findings of this study suggest that the arch support function of the sock may attenuate inefficient propulsion during flatfoot gait. Further verification of the direction of these hypotheses will require large-scale sample studies in the future. Because this study relied exclusively on EMG measures, the observed reduction in muscle activity should not be interpreted as direct evidence of improved gait efficiency or neuromuscular efficiency; such an interpretation would require corroborating kinematic and kinetic data, which were not collected in this study.

The bilateral asymmetry in the observed EMG changes is consistent with the right-dominant composition of the sample, as discussed in Section 4.2. Future studies should employ stratified analyses based on dominant foot status.

Given the excellent measurement reliability confirmed over all the eight channels, the failure of the observed changes to meet MDC95 thresholds is unlikely to reflect measurement instability. Instead, it is more consistent with the inherent magnitude constraints of single-session interventions.

From a clinical standpoint, the immediate neuromuscular changes showed in this study provide preliminary evidence that sock-type arch-supportive devices may beneficially affect gait mechanics in adults with flatfoot. However, rigorous clinical validation will require longitudinal studies over varying flatfoot severities and functional activity conditions.

### 4.4. Strengths

A primary methodological strength of this study is the high degree of measurement reliability. ICC(3,1) values of 0.95 or above were obtained over all eight channels, which meet the threshold for excellent within-trial reliability according to Koo and Li [27]. This supports the interpretation that the observed pre-to-post changes are unlikely to be attributable to measurement variability alone; however, given the single-arm design, alternative explanations such as measurement repetition, learning effects, or short-term neuromuscular adaptation cannot be fully excluded.

A second strength is the rigorous adherence to established methodological standards. Bilateral simultaneous eight-channel recordings enabled within-session pre-to-post comparisons and between-limb analysis of the sock effects. The EMG amplitudes were normalized to %MVIC [19], and parametric and non-parametric tests were selected for each channel based on normality testing. Moreover, the effect sizes (Cohen’s d; Wilcoxon r) were determined for all the comparisons to support interpretation beyond statistical significance alone.

Finally, to our knowledge, this is the first study to examine the neuromuscular effects of a TPU-integrated sock-type arch-supportive device. This approach prioritizes daily-life compatibility over the prescription-dependent framework of conventional insoles and orthoses. The eight-channel EMG dataset generated in this study constitutes foundational reference data for subsequent investigations into long-term effects and clinical applicability of sock-type assistive devices.

### 4.5. Limitations

The primary limitation of this study is its single-session design, which precludes evaluation of cumulative or long-term effects. As mentioned in Section 3, the observed changes in all five significant channels are below their respective MDC95 thresholds. This finding is consistent with the inherent magnitude constraints of single-session interventions. Therefore, longitudinal studies are required to determine whether extended sock use produces changes of clinical significance. Furthermore, this study employed a single-arm pre–post design without a control group; therefore, the observed changes cannot be fully attributed to the TPU arch pad sock alone, and contributions from measurement repetition or short-term neuromuscular adaptation cannot be excluded. Future studies should incorporate a control condition (e.g., a sham sock or standard sock without the TPU pad) to isolate the specific effect of the arch pad.

The second limitation relates to the sample size. This study was conducted in adults with flexible flatfoot (ten males and ten females), and a priori sample size calculation (power analysis) was not performed. As this study adopted an exploratory design without a priori power analysis, the sample size (*n* = 20) was determined based on feasibility considerations rather than statistical power estimation [13]; this may limit the precision of the effect size estimates and the generalizability of the findings. Furthermore, because this study was conducted exclusively in adults with flexible flatfoot, generalizability to various flatfoot types including severe and rigid flatfoot is limited. Furthermore, participants were not stratified by the presence or severity of flatfoot-related symptoms (e.g., pain or fatigue); therefore, the generalizability of these findings to clinically symptomatic individuals with flexible flatfoot remains to be established. In addition, the sample was restricted to young adults aged 20–29 years; therefore, the findings may not generalize to older individuals, who may exhibit different neuromuscular or gait characteristics. Therefore, the generalizability of the present findings may be expanded if the limitations of sample size are addressed by large-scale follow-up studies including various flatfoot severities.

The third limitation arises from the methodological aspects. The navicular drop test for flatfoot diagnosis was performed only on the dominant foot of each participant. Flatfoot presentation of the non-dominant foot was confirmed solely by participant self-report, without quantitative assessment. Furthermore, because all the measurements were performed by a single trained researcher, inter-rater reliability was not assessed. However, the ICC values over all the eight channels were excellent in this study, thereby suggesting the measurement consistency of the single-rater design. In future studies, these methodological limitations may be addressed by quantitative bilateral flatfoot diagnosis and inter-rater reliability assessment using a multi-rater design. Tibialis posterior, peroneus brevis and the intrinsic foot muscles, although relevant to arch support, were not included primarily due to technical limitations of surface EMG: tibialis posterior lies deep to other musculature and is difficult to isolate without indwelling electrodes, and the intrinsic foot muscles are similarly inaccessible to reliable surface recording. Peroneus brevis was excluded due to its close anatomical proximity to peroneus longus, increasing crosstalk risk.

Electrode crosstalk between adjacent muscles (e.g., medial and lateral gastrocnemius, and the peroneal muscle group) was minimized via SENIAM-recommended electrode placement over the muscle belly [18]; however, quantitative crosstalk assessment (e.g., cross-correlation analysis) was not performed, and this is noted as a methodological limitation.

In addition, the pad position was identical across all the participants and was based on standard anatomical landmarks. However, discrepancies between the applied pad position and the actual navicular tuberosity location may exist due to individual variations in foot anatomy. Future studies should consider individualized pad placement reflecting individual foot morphology.

Furthermore, considering the design characteristics of immediate effect assessment, a unidirectional measurement order of pre → post was applied. Order and learning effects were therefore not controlled. This should be considered as a methodological limitation of this study. A randomized or counterbalanced measurement order, or the inclusion of a sham-controlled condition, should be considered in future studies to control for order and learning effects; this limitation should be regarded as a substantial constraint on causal interpretation rather than a minor methodological caveat.

Self-selected walking speed was adopted to maximize ecological validity, and the within-subject design was expected to minimize intra-session speed variation. However, walking speed was not quantitatively recorded under the pre and post conditions.

Because walking velocity is known to influence lower-extremity EMG activity, changes in self-selected speed between the pre- and post-intervention conditions may have partially contributed to the observed differences in muscle activity; accordingly, this should be regarded as an important potential confounding factor rather than a minor measurement limitation.

The fourth limitation is that the TPU material used in the sock was of general industrial grade rather than medical grade. Because this study was designed for short-term immediate effect measurement aimed at reducing muscle activity and improving gait patterns in adults with flexible flatfoot, the use of medical-grade material was not essential. Thus, industrial-grade TPU with confirmed safety by preliminary safety review and pilot testing was used. However, differences in the safety and efficacy of medical- and industrial-grade materials have not been evaluated in the context of long-term sock use. Therefore, comparative studies utilizing medical-grade TPU should be conducted to evaluate material grade-dependent differences in the future. Independent mechanical characterization of the TPU arch pad (e.g., stiffness, durability under cyclic loading, and plantar-pressure distribution) was not performed in this study. Such characterization represents an important priority for future device validation studies prior to clinical application. The present EMG-only design cannot distinguish whether the observed reduction in muscle activity reflects improved neuromuscular efficiency or a genuine reduction in muscular contribution to foot and ankle stabilization. Combined kinematic and kinetic measurements (e.g., joint angle, ground reaction force, or center-of-pressure trajectory) in future studies would be necessary to differentiate between these possibilities. Future studies should incorporate additional functional outcome measures, such as plantar pressure distribution, spatiotemporal gait parameters, or patient-reported outcomes, to more comprehensively evaluate the clinical relevance of the TPU arch pad sock.

This study has the aforementioned limitations in terms of single-session intervention design, sample size, measurement methodology, and material grade. However, it holds academic and clinical significance. It provides initial evidence that sock-type arch-supportive assistive devices may affect lower-extremity muscle activity in adults with flexible flatfoot.

In future follow-up studies, the findings of this study should be more rigorously verified. Such studies should address the aforementioned limitations, including research design, sample size, quantitative diagnosis, rater design, measurement order and speed settings, and material grade.

## 5. Conclusions

In this study, the immediate effects of a TPU arch pad sock application on lower-extremity EMG activity were evaluated in 20 adults with flexible flatfoot.

Statistically significant reductions in muscle activity were observed in five of the eight channels following the sock application, with large effect sizes confirmed in the right TA, right PL, and right MG.

The reduction in MG activity was observed in the direction opposite to the a priori hypothesis. This suggests that arch support delivered by a sock-type assistive device may attenuate the compensatory neuromuscular burden associated with inefficient propulsion during flatfoot gait.

However, the observed changes did not exceed the MDC95 for any of the five significant channels, indicating that these immediate effects did not reach the level of clinically meaningful change considering the inherent characteristics of single-session measurements. As this study was exploratory in nature and was not powered a priori, these findings should be regarded as preliminary. Therefore, adequately powered, large-scale follow-up studies including various flatfoot severities are needed to confirm the reported effects and establish the cumulative effects of long-term use and clinical efficacy.

If the cumulative effects of long-term TPU arch pad sock use and clinical efficacy are established by future follow-up studies, the TPU arch pad sock may represent a potential approach for improving inefficient gait patterns in adults with flatfoot in daily life, pending confirmation of long-term clinical efficacy in future controlled studies.

## Figures and Tables

**Figure 1:**
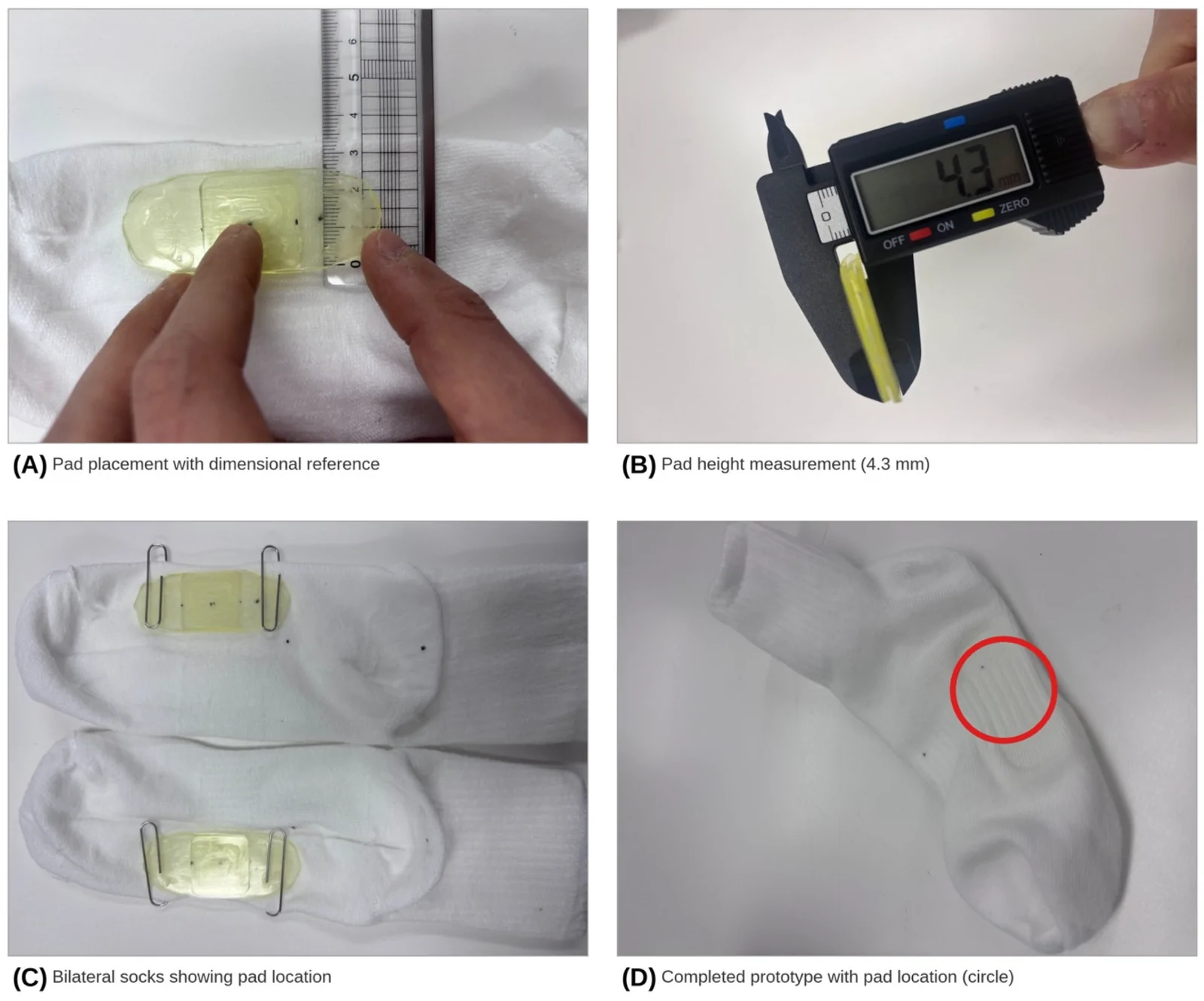
TPU arch pad sock prototype. (**A**) Pad placement with dimensional reference; (**B**) Pad height measurement (4.3 mm); (**C**) Bilateral socks showing pad location; (**D**) Completed prototype with pad location (circle).

**Figure 2:**
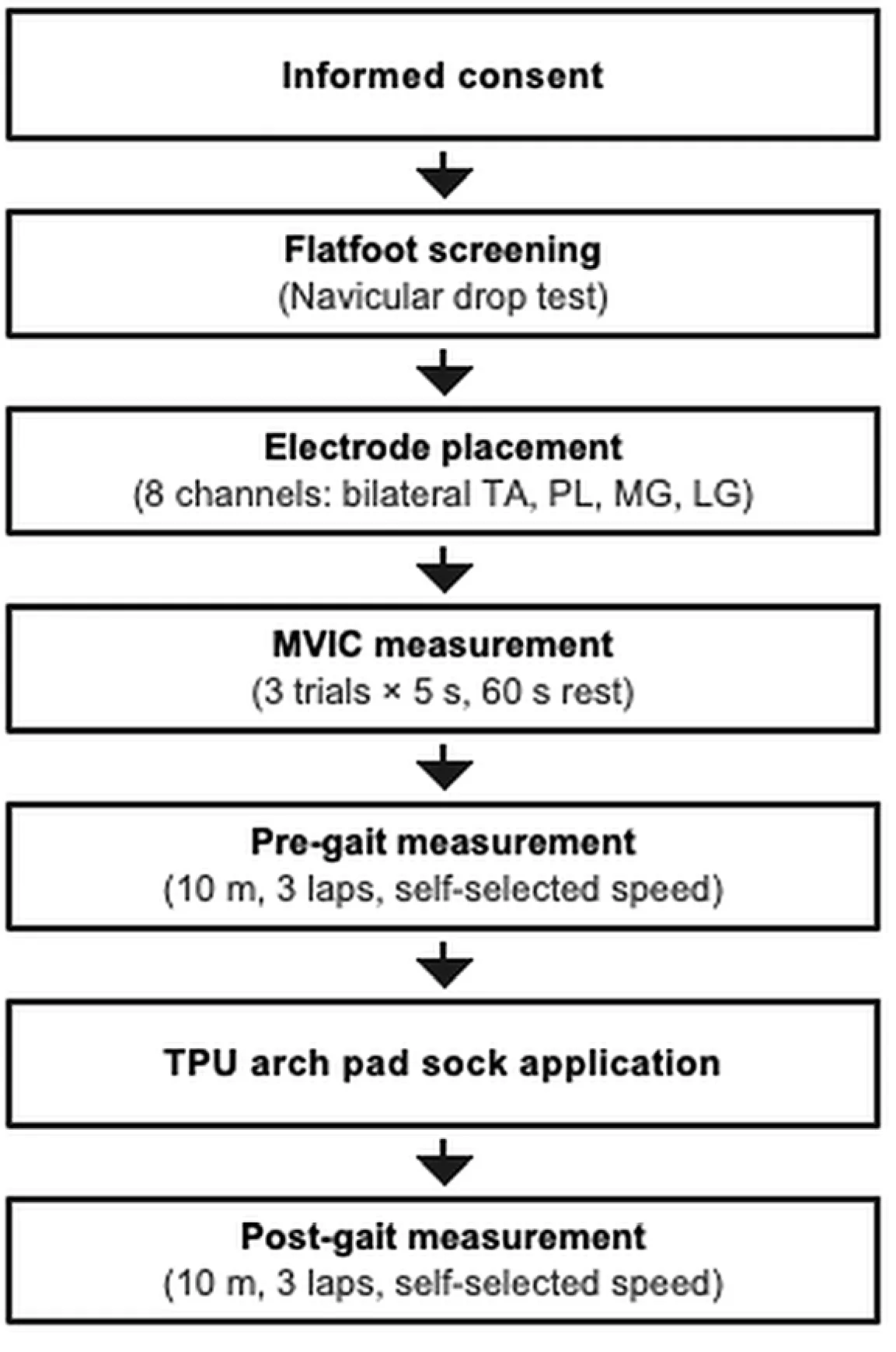
Experimental procedure flowchart.

**Figure 3:**
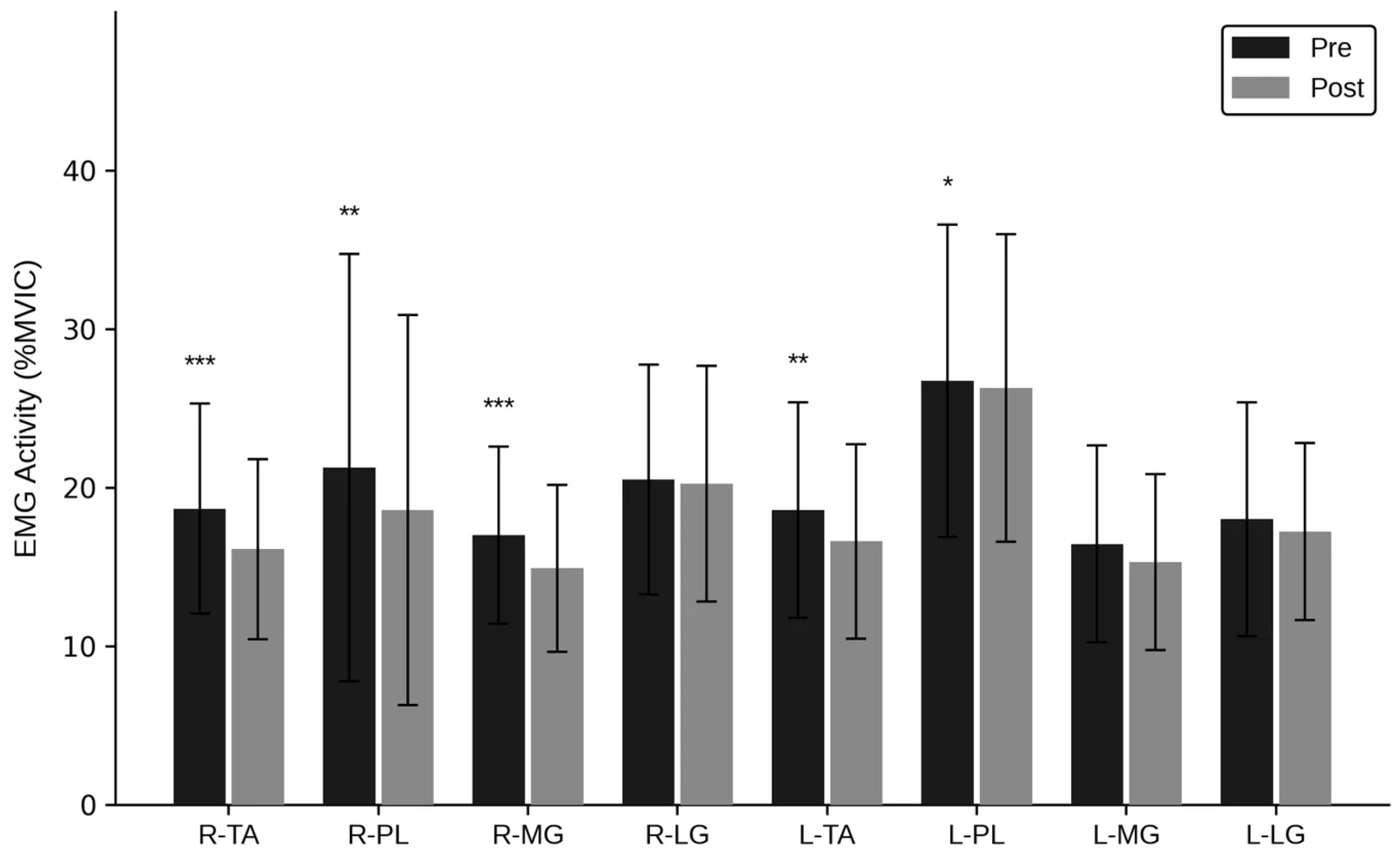
Comparison of the pre- and post-intervention EMG activities over all the channels (mean ± SD). * *p* < 0.05, ** *p* < 0.01, *** *p* < 0.001.

**Table 1:** Participant demographic characteristics (*n* = 20).

Variable	Mean ± SD	Range
Sex (Male/Female)	10/10	
Age (years)	25.4 ± 2.4	
Height (cm)	167.6 ± 6.7	
Weight (kg)	66.6 ± 12.9	
BMI (kg/m^2^)	23.60 ± 3.60	
Foot length (mm)	251.5 ± 14.9	
Dominant foot (Right/Left)	17/3 (85%/15%)	
NWB navicular height (mm)	41.15 ± 6.16	
WB navicular height (mm)	28.80 ± 5.76	
Navicular drop (mm)	12.35 ± 2.41	10.00–19.00

BMI, body mass index; NWB, non-weight-bearing; WB, weight-bearing.

**Table 2:** Pre- and post-intervention EMG activity and statistical results (*n* = 20).

Channel	Pre (%MVIC)	Post (%MVIC)	W	*p*(S-W)	Test	Statistic	*p*	*p* (FDR)	Effect Size
R-TA	18.68 ± 6.63	16.13 ± 5.69	0.639	<0.001	Wilcoxon	Z = −4.130	<0.001 ***	<0.001 ***	r = 0.924
R-PL	21.27 ± 13.47	18.60 ± 12.31	0.926	0.127	*t*-test	t = 4.031	0.001 **	0.002 **	d = 0.901
R-GCM	17.01 ± 5.60	14.93 ± 5.27	0.919	0.095	*t*-test	t = 5.175	<0.001 ***	<0.001 ***	d = 1.157
R-GCL	20.52 ± 7.26	20.25 ± 7.44	0.868	0.011	Wilcoxon	Z = −0.384	0.701	0.801	r = 0.086
L-TA	18.60 ± 6.81	16.63 ± 6.13	0.936	0.198	*t*-test	t = 3.064	0.006 **	0.013 *	d = 0.685
L-PL	26.75 ± 9.84	26.30 ± 9.69	0.843	0.004	Wilcoxon	Z = −2.166	0.030 *	0.049 *	r = 0.484
L-GCM	16.45 ± 6.21	15.32 ± 5.54	0.926	0.130	*t*-test	t = 1.241	0.230	0.306	d = 0.278
L-GCL	18.02 ± 7.39	17.24 ± 5.58	0.627	<0.001	Wilcoxon	Z = −0.055	0.956	0.956	r = 0.012

Values are mean ± SD. W, Shapiro–Wilk statistic; S-W, Shapiro–Wilk test; d, Cohen’s d; r, Wilcoxon effect size. *p* (FDR), *p*-value adjusted for multiple comparisons across the eight channels using the Benjamini–Hochberg false discovery rate procedure [22]. *p*(S-W) denotes the *p*-value of the Shapiro–Wilk normality test performed on the pre-post difference (delta) for each channel, distinct from the *p*-value of the subsequent paired *t*-test or Wilcoxon signed-rank test reported in the ‘*p*’ column.

**Table 3:** Measurement reliability of EMG activity across all channels (*n* = 20).

Channel	ICC(3,1) [Pre Only]	ICC(3,1) [Pre + Post Combined]
R-TA	0.969	0.883
R-PL	0.976	0.972
R-MG	0.980	0.950
R-LG	0.978	0.966
L-TA	0.958	0.904
L-PL	0.996	0.989
L-MG	0.965	0.816
L-LG	0.980	0.868

ICC, intraclass correlation coefficient; EMG, electromyography. The pre-only ICC represents the primary reliability estimate, reflecting measurement consistency under baseline conditions. The pre + post combined ICC is provided as a sensitivity analysis; because the intervention was expected to produce genuine pre-to-post change, this combined estimate is conceptually conservative and should not be interpreted as evidence of reduced measurement reliability.

**Table 4:** Comparison of the mean change (|D|) and MDC95 for the statistically significant channels.

Channel	|Δ| (%MVIC)	MDC95 (%MVIC)	Interpretation
R-TA	2.54	3.26	Below MDC95
R-PL	2.67	5.70	Below MDC95
R-MG	2.08	2.20	Below MDC95
L-TA	1.97	3.96	Below MDC95
L-PL	0.45	1.64	Below MDC95

**Table 5:** Comparison of the present study with previous studies examining lower-extremity EMG activity in adults with flatfoot.

Study	*n*	Intervention	Muscles Assessed	Key Finding	Direction
Murley et al. (2010) [8]	30 (flat-arched)	Prefabricated vs. customised foot orthosis	TP, PL (indwelling); TA, MG (surface)	TP activity decreased 19% (prefabricated) and 12% (customised) vs. shoe-only during the contact phase (*p* < 0.008)	Consistent (↓)
Scott et al. (2012) [29]	28 (flat-arched)	Barefoot vs. standard shoe vs. stability running shoe	TP, PL (indwelling); TA, MG (surface)	Significant differences in peak amplitude for TA, PL, and MG across conditions (*p* < 0.05)	Consistent (muscle overlap)
Huang et al. (2017) [30]	16 (flatfoot)	Arch-support vs. flat insole	GAS, TA, BF, RF (surface)	RF median frequency slope improved with arch-support insole during downhill walking (reduced fatigue)	Consistent (different metric)
**Present study**	**20 (flexible flatfoot)**	**TPU arch pad sock**	**TA, PL, MG, LG (bilateral, surface)**	**Significant %MVIC reduction in R-TA, R-PL, R-MG, L-TA, and L-PL (5 of 8 channels)**	**—**

TP, tibialis posterior; PL, peroneus longus; TA, tibialis anterior; MG, medial gastrocnemius; LG, lateral gastrocnemius; GAS, gastrocnemius (head not specified in the original study); BF, biceps femoris; RF, rectus femoris. Huang et al. [30] assessed muscle fatigue (median frequency) rather than activity amplitude (%MVIC); this metric difference is noted because it precludes direct numerical comparison with the present study.

## Data Availability

The data presented in this paper are available upon reasonable request from the corresponding author.
